# The Effects of Varying Concentrations of Dietary Protein and Fat on Blood Gas, Hematologic Serum Chemistry, and Body Temperature Before and After Exercise in Labrador Retrievers

**DOI:** 10.3389/fvets.2016.00059

**Published:** 2016-08-02

**Authors:** John Ober, Robert L. Gillette, Thomas Craig Angle, Pamela Haney, Daniel J. Fletcher, Joseph J. Wakshlag

**Affiliations:** ^1^Department of Clinical Sciences, Cornell University College of Veterinary Medicine, Ithaca, NY, USA; ^2^Animal Health and Performance Center, Auburn University College of Veterinary Medicine, Auburn, AL, USA

**Keywords:** dietary fat, lactate, protein, fat, performance, body temperature

## Abstract

Optimal dietary protocols for the athletic canine are often defined by requirements for endurance athletes that do not always translate into optimal dietary interventions for all canine athletes. Prior research studying detection dogs suggests that dietary fat sources can influence olfaction; however, as fat is added to the diet the protein calories can be diminished potentially resulting in decreased red blood cell counts or albumin status. Optimal macronutrient profile for detection dogs may be different considering the unique work they engage in. To study a calorically low protein: high fat (18:57% ME), high protein: high fat (27:57% ME), and high protein: low fat (27:32% ME) approach to feeding, 17 dogs were provided various diets in a 3 × 3 cross over design. Dogs were exercised on a treadmill and blood was taken pre-exercise, immediately post-exercise, 10- and 20-min post-exercise to assess complete blood count, serum chemistry, blood gases, and cortisol; as well as rectal and core body temperature. Exercise induced a decrease in serum phosphorus, potassium, and increases in non-esterified fatty acids and cortisol typical of moderate exercise bouts. A complete and balanced high protein: high-fat diet (27:57% ME) induced decreases in serum cortisol and alkaline phosphatase. Corn oil top dressed low protein: high-fat diet (18:57% ME) induced a slightly better thermal recovery than a complete and balanced high protein: high fat diet and a high protein: low fat (27%:32% ME) diet suggesting some mild advantages when using the low protein: high fat diet that warrant further investigation regarding optimal protein and fat calories and thermal recovery.

## Introduction

Research surrounding optimal feeding patterns for the canine athlete has been investigated since the early 1930s with significant advances in feeding endurance canine athletes (1–3). Considerable debate regarding the optimal amount of fat and protein needed to fuel the canine athlete continues; however, Kronfeld and colleagues suggest that carbohydrate is not necessary in the endurance dog diet (4). Downey further clarified this idea studying beagles on treadmills showing that stamina improved when utilizing higher fat diets with approximately 69% metabolizable energy (ME) as fat versus 27% ME (5). Later observations showed that protein needed to be approximately 32% ME for endurance huskies to maintain their red blood cell volume for the duration of a training and racing season (6) with follow up studies examining sprint racing huskies suggesting that approximately 24% ME from protein was required to maintain optimal performance (7). Greyhound studies are less clear with consensus being that protein should be around 24% ME or higher (8). This has led to firm recommendations that performance dogs be fed over 24% ME as protein for all performance parameters to maintain red cell mass, maintain serum albumin status and promote less musculoskeletal injury; while the remaining caloric intake come from fat or carbohydrate depending on the athletic endeavor (1–3).

Even less is known about the needs of hunting and detection dogs whose primary function is olfactory activity. Many trainers and hunters provide high quality commercial dog foods with a higher fat content adopting much of the nutritional dogma of endurance sled dogs. An interesting study examined performance of plantation hunting pointers on two dog foods with similar nutrient profiles, differing minimally in protein (24–26% ME) and more from fat (10% ME difference), which showed that finds per hour were significantly lower when dogs were on the lower fat diet suggesting that a change in commercial diet can influence detection (9). Further work examining fat intake and olfaction showed that polyunsaturated fatty acids improved or maintained the efficiency of olfaction in trained pointers, while dogs fed coconut oil showed a loss of olfactory acuity (10). These findings are similar to rodent studies which suggested that polyunsaturated fatty acids may affect olfaction positively by altering the olfactory bulb cellular constitution, thereby enhancing neuronal signaling (11, 12). More recently, our lab reported that the use of corn oil improved the olfactory acuity at threshold concentrations in trained detection dogs showing that dietary fat and fat source can play a role in olfaction and/or cognition, yet the physiology of such improvements can only be speculated (13). Theoretically, dietary fat may offer an advantage of running with more thermodynamic efficiency compared to a higher protein, higher carbohydrate diets since there is less of a need for gluconeogenesis during exercise eliminating protein as a source of energy which is thermodynamically less efficient than fat or carbohydrate (6). Contradictory results suggest that glucose helps decrease thermal load by improving respiratory dynamics and loss of heat through panting (14). Based on contradictory theories, our objectives were to utilize the same Labrador Retrievers used in our previous olfaction study, to examine the effects of diet and exercise on serum lactate, blood gases, cortisol response, complete blood counts, serum biochemistry, and body temperature (rectal and core body temperature), to better understand the physiology that might explain enhanced olfaction when fed a corn oil-supplemented diet (13). Seventeen detection dogs on two standard commercial dog foods that consisted of either low fat (LF – 27% ME protein, 32% ME fat) or high-fat performance diet (HF – 27% ME protein, 57% ME fat); as well as a low protein food with high fat made through top dressing a typical adult maintenance diet with corn oil (CO – 18% ME protein, 57% ME fat) in a cross-over design before and after exercise.

## Materials and Methods

### Animal Housing and Feeding

The use of animals in this study was approved by the Auburn University Animal Care and Use Committee. All dogs were housed in a 1.3 m × 2.6 m enclosure, which had an attached 1.3 m × 4 m outdoor facility that was available to the dogs daily. Seventeen sexually intact Labrador Retrievers (11 males and 7 females) between the ages of 18 and 24 months were enrolled in the study, with one male dog being discontinued within 4 weeks of starting the study due to an acute cranial cruciate tear. Throughout each 12-week “arm” of each treatment dogs were fed either a normal maintenance diet^1^ [Royal Canin Maintenance; 27% ME protein, 32% ME as fat, 41% ME as carbohydrate (LF)], a performance diet^2^ [27% ME protein, 57% ME fat, 14% ME carbohydrate (HF)], or a maintenance diet (see text footnote 1) with additional corn oil^3^ [14.5 g of corn oil per cup of kibble fed at 269 kcals/cup; 18% ME protein, 57% ME fat, 25% ME carbohydrate (CO)]. Grams of protein intake per kg body weight for each dietary group median and range was 4.5 g/kg (3.8–6.2 g/kg) in the LF feeding, 4.4 g/kg (3.7–6.0 g/kg) when consuming the HF diet and 3.1 g/kg (2.7–4.4 g/kg) when consuming the CO diet. Each dog was fed to maintain body condition between a body condition score of 4 or 5 out of 9 and to maintain body weight from the beginning of the feeding trial until the end with kilocalorie consumption never varying more than ±15% of the caloric intake at the beginning of the dietary trial. Dog weights were recorded each week, and diets were adjusted to ensure that that the dogs did not lose or gain more than 5% of their body weight.

### Conditioning Protocol

All dogs were trained and conditioned for detection performance activities by road training (30 min on the end of a rod attached to a golf cart) and 30 min of scent-detection training (smokeless powder, ammonium nitrate, and trinitrotoluene in decreasing concentrations) five times per week. All dogs were acclimated to treadmill by running 15–20 min once a week during their training and a conditioning regimen that was approximately 6 months prior to the dietary study. All dogs were between 2 and 3 years of age and had been trained for detection activity in the urban and rural field settings. Three months prior to the study, all dogs were fed a typical maintenance ration (see text footnote 1). Dogs were then randomly drawn from a hat and partitioned into three dietary groups. Conditioning and training protocols remained the same for all dogs during the dietary trials each of which lasted 12 weeks; thus, each dog served as its own control during the dietary trials.

### Exercise Test

To examine the effects of diet and exercise, at the end of each 12-week arm, dogs ran on a stationary treadmill for 30 min daily at a 2.5% incline at 12.5 km/h on three consecutive days.^4^ Standard commercial fans were mounted at eye level with the dogs, just to the left of the treadmills and put on medium settings (3000 CFU) to simulate wind rushing across the nasal and oral cavities. The rooms where the dogs ran were kept at 30% humidity and 72°F at all times. All data and sample collection occurred on day 3 of the 3 days of treadmill testing on week 12, while olfaction data were obtained on the other 2 days of exercise (13).

### Rectal and Core Temperature Monitoring

Before running, the dogs’ body temperature was assessed through core body temperature and rectal temperature monitoring. Core body temperature was assessed by administering a small thermistor pill^5^ 20 min before the exercise bout and temperatures were taken immediately before exercise and then every 5 min during exercise, and immediately after exercise as well as 10 and 20 min intervals during recovery in tandem with rectal temperature acquisition. Rectal temperatures were assessed using the Becton Dickenson digital thermometer.^6^ All readings were made approximately 1 min prior to treadmill exercise, immediately after coming off of the treadmill, and then again 10 and 20 min into recovery post-exercise.

### Blood and Serum Collection and Analysis

Jugular venipuncture was performed on the third day at the aforementioned time intervals (pre, post, 10 min after, and 20 min after) for assessment of blood gases, complete blood count (including: red blood cells, hematocrit, hemoglobin, white blood cells, neutrophils, and lymphocytes), serum biochemistry [including; sodium, potassium, phosphorus, calcium, magnesium, chloride, albumin, globulin, urea nitrogen, creatinine, alanine amino transferase (ALT), aspartate amino transferase (AST), creatine kinase, non-esterified fatty acid, glucose], and cortisol concentrations. Venous blood gas analysis was performed on iSTAT analyzer^7^ using the CG8^+^ cartridge^8^ within 2 min of sample collection. The remaining sample was aliquoted into a coagulation tube or EDTA tube and refrigerated for approximately 30 min until centrifuged at 4,000 *g* for 10 min for serum collection. EDTA collected blood was shipped overnight on ice packs to Antech laboratory and analyzed using an Advia 120 cell counter.^9^ Separated serum was frozen at −20°C and within a week all samples were shipped on dry ice overnight to the Animal Health Diagnostic Laboratory at Cornell University for analysis on a Hitachi 911 analyzer.^10^ Serum cortisol concentrations were analyzed using validated canine radioimmunoassay at the Cornell University Diagnostic Laboratory Endocrinology Unit.

### Statistical Analysis

To account for the study design, a mixed model analysis of variance with the fixed effects of time, diet, period of treatment, and sequence of treatment was chosen. The interaction of time and diet was forced into every model. Random effects in the model were ID and period nested within ID. Residuals were examined for normality. In cases were residuals were non-normally distributed, a log transformation of the dependent variable was successful to satisfy the model assumptions. *P*-values are reported for the fixed effects of time, diet, and diet × time. If the *P*-value for the effect of time or diet reached significance (*P* smaller 0.05), a Dunnett’s *post hoc* analysis was performed to examine the strength of association from pre-exercise or the low-fat diet, controlling for multiple comparisons. Significance was set at an alpha of 0.05. JMP 12.0^11^ was used for all statistical analyses, and Graph pad Prism 6.0^12^ was used for graphing.

## Results

### Complete Blood Count

There were no significant interactions of diet and exercise for red blood cells (*P* = 0.96), hematocrit (*P* = 0.99), hemoglobin (*P* = 0.97), white blood cells (*P* = 0.61), neutrophils (*P* = 0.97), or lymphocytes (*P* = 0.71) (Table 1).

**Table 1:** **Mean and SD for complete blood count pre and post exercise; 10-min post exercise and 20-min post exercise for three dietary groups (*n* – 17) during feeding a high-fat high-protein diet (HF), high corn oil fat low-protein diet (CO), and a low-fat high-protein diet (LF)**.

CBC (ref. range)	Diet	Pre	Post	Post 10 Min	Post 20 Min	Time	Diet	Time × Diet
RBC (millions/mL) (4.8–9.3)	HF	6.6 ± 0.4	**7.1 ± 0.3**	6.6 ± 0.4	6.5 ± 0.4	*P* < 0.01	*P* < 0.01	*P* = 0.96
	CO^a^	6.5 ± 0.6	**6.9 ± 0.6**	6.5 ± 0.5	6.4 ± 0.5			
	LF	6.6 ± 0.4	**7.1 ± 0.5**	6.7 ± 0.5	6.5 ± 0.5			
HCT (%) (36–60)	HF	49.1 ± 3.0	**52.1 ± 2.4**	49.2 ± 3.6	48.8 ± 3.1	*P* < 0.01	*P* = 0.16	*P* = 0.99
	CO	48.5 ± 3.8	**50.9 ± 3.0**	47.8 ± 4.1	47.3 ± 3.9			
	LF	48.4 ± 3.9	**51.5 ± 3.3**	48.8 ± 3.9	47.9 ± 4.6			
Hemoglobin (g/L) (12.1–20.3)	HF	15.6 ± 0.8	**16.5 ± 0.8**	15.4 ± 1.0	15.2 ± 1.0	*P* < 0.01	*P* = 0.04	*P* = 0.97
	CO^a^	15.3 ± 1.2	**16.0 ± 1.1**	15.0 ± 1.1	15.0 ± 1.1			
	LF	15.3 ± 0.9	**16.3 ± 1.2**	15.4 ± 1.1	15.1 ± 1.1			
WBC (thousands/mL) (4–15.5)	HF	9.5 ± 2.4	9.2 ± 2.6	9.2 ± 2.4	**9.0 ± 2.4**	*P* = 0.04	*P* < 0.01	*P* = 0.61
	CO^a^	8.8 ± 1.9	8.7 ± 2.1	8.9 ± 1.7	**8.4 ± 2.0**			
	LF	9.7 ± 1.7	8.7 ± 1.9	8.9 ± 1.7	**9.0 ± 1.8**			
Neutrophils (2060–10600)	HF	6003 ± 1972	6292 ± 2086	6103 ± 1988	6026 ± 1954	*P* < 0.48	*P* = 0.01	*P* = 0.97
	CO^a^	5557 ± 1280	5831 ± 1580	5839 ± 1336	5477 ± 1428			
	LF	5949 ± 1296	5823 ± 1363	5826 ± 1284	5694 ± 1297			
Lymphocytes (690–4500)	HF	2406 ± 718	**2028 ± 622**	**2167 ± 603**	**2099 ± 675**	*P* < 0.01	*P* = 0.24	*P* = 0.71
	CO	2368 ± 841	**2039 ± 724**	**2150 ± 588**	**2118 ± 710**			
	LF	2611 ± 510	**1967 ± 537**	**2221 ± 476**	**2311 ± 520**			

*Bolded text indicates a difference from pre-exercise over time; ^a^In dietary groups represents a significant difference between groups (*P* < 0.05)*.

Dietary differences were not observed for HCT (*P* = 0.16) or lymphocytes (*P* = 0.24). There were dietary decreases observed for the CO group compared to the LF and HF groups for red blood cells (*P* < 0.01), hemoglobin (*P* = 0.04), white blood cells (*P* < 0.01), and neutrophils (*P* = 0.01).

There were significant differences observed across time for hematocrit (*P* < 0.01), red blood cells (*P* < 0.01), hemoglobin (*P* < 0.01), white blood cells (*P* = 0.04), and lymphocytes (*P* < 0.01). Numerically, red blood cell and HCT concentrations increased post-exercise due to mild hemoconcentration; while lymphocytes decreased post-exercise and remained lower during recovery (*P* < 0.01). This was also reflected in a lower total white blood cell count at 20 min of recovery (*P* = 0.04).

### Venous Blood Gas Analysis

There were no interactions between diet and exercise observed for base excess (*P* = 0.17), pvCO_2_ (*P* = 0.10), TCO_2_ (*P* = 0.16), bicarbonate (*P* = 0.19), and pH (*P* = 0.17) (Table 2).

**Table 2:** **Mean and SD for blood gases pre and post exercise; 10-min post exercise and 20-min post exercise for three dietary groups (*n* – 17) during feeding a high-fat high-protein diet (HF), high corn oil fat low protein diet (CO), and a low-fat high-protein diet (LF)**.

	Diet	Pre	Post	Post 10 Min	Post 20 Min	Time	Diet	Diet × time
Base Exc. (mmol/L)	HF	−2.5 ± 1.5	**−4.5 ± 1.3**	**−5.4 ± 1.5**	**−5.2 ± 1.7**	*P* < 0.01	*P* < 0.16	*P* = 0.17
	CO	−2.2 ± 1.7	**−4.0 ± 1.7**	**−4.5 ± 1.5**	**−4.3 ± 1.8**			
	LF	−1.6 ± 1.6	**−4.1 ± 1.9**	**−5.0 ± 2.0**	**−4.8 ± 2.1**			
pCO_2_ (mmHg)	HF	33.9 ± 3.4	**22.3 ± 6.0**	**24.9 ± 6.1^b^**	**28.4 ± 5.8**	*P* < 0.01	*P* < 0.01	*P* < 0.10
	CO^a^	35.0 ± 3.3	**21.7 ± 4.9**	**27.7 ± 4.6^a^**	**30.3 ± 3.8**			
	LF	35.1 ± 5.0	**20.3 ± 7.7**	**23.8 ± 6.5^b^**	**29.0 ± 5.0**			
TCO_2_ (mmHg)	HF	22.9 ± 1.9	**18.8 ± 2.3**	**18.7 ± 2.4**	**19.7 ± 2.4**	*P* < 0.01	*P* < 0.28	*P* < 0.16
	CO	23.4 ± 2.1	**19.1 ± 2.0**	**20.2 ± 1.9**	**20.8 ± 2.0**			
	LF	23.8 **±** 2.0	**18.6 ± 3.5**	**19.0 ± 3.1**	**20.2 ±2.4**			
HCO3 (mmol/L)	HF	22.0 ± 1.7	**18.1 ± 2.1**	**18.1 ±2.2**	**18.9 ±2.1**	*P* < 0.01	*P* = 0.22	*P* = 0.19
	CO	22.5 ± 1.9	**18.4 ± 1.9**	**19.4 ±1.8**	**19.9 ±1.8**			
	LF	22.8 ± 1.8	**17.9 ±3.3**	**18.2 ± 2.9**	**19.4 ± 2.2**			
pH	HF	7.42 ± 0.02	**7.53 ±0.09**	**7.48 ± 0.07**	7.44 ± 0.06	*P* < 0.01	*P* = 0.11	*P* = 0.17
	CO	7.42 ± 0.02	**7.55 ± 0.08**	**7.46 ± 0.05**	7.43 ± 0.02			
	LF	7.43 ± 0.04	**7.58 ± 0.12**	**7.50 ± 0.06**	7.44 ± 0.05			

*Bolded text indicates a difference from pre-exercise over time; ^a^In dietary groups represents a significant difference between groups (*P* < 0.05). ^a,b^Across time points represent significant differences (*P* < 0.05) between groups at individual times with similar or no superscripts signifying no differences*.

No effects of diet were observed on blood gases other than an overall increased pvCO_2_ (*P* = 0.01). Dogs consuming the CO diet had significantly higher pvCO_2_ 10-min post-exercise than dogs consuming the LF diet or HF diet (*P* = 0.02).

Immediately after exercise, the pvCO_2_ was lower immediately post-exercise, and 10-min post exercise, and returned to pre-exercise tensions at 20-min post exercise (*P* < 0.01). Blood base excess and bicarbonate concentrations were also lower immediately post-exercise and remained lower than pre-exercise values at 10- and 20-min post-exercise (*P* < 0.05). Following the exercise bout, all groups had significantly higher blood pH (*P* < 0.05), which recovered over time, approaching pre-exercise values by 20-min post-exercise.

### Serum Chemistry

There were no significant diet or exercise interactions observed (all values *P* > 0.1) (Table 3).

**Table 3:** **Mean and SD for serum biochemistry pre and post exercise; 10-min post-exercise and 20-min post-exercise for three dietary groups (*n* – 17) during feeding a high-fat high-protein diet (HF), high corn oil fat low protein diet (CO), and a low-fat high-protein diet (LF)**.

Serum Biochem (Ref. range)	Diet	Pre	Post	Post 10 Min	Post 20 Min	Time	Diet	Diet × time
Sodium (mEq/L) (142–151)	HF	147 ± 2	148 ± 2	148 ± 2	**149 ± 2**	*P* < 0.01	*P* = 0.67	*P* = 0.91
	CO	148 ± 3	147 ± 2	148 ± 2	**149 ± 2**			
	LF	147 ± 2	148 ± 2	148 ± 2	**149 ± 2**			
Chloride (mEq/L) (107–117)	HF^a^	113 ± 2	114 ± 2	**115 ± 2**	**115 ± 2**	*P* < 0.01	*P* < 0.01	*P* = 0.36
	CO	112 ± 2	114 ± 2	**115 ± 2**	**115 ± 2**			
	LF	112 ± 2	114 ± 2	**114 ± 2**	**115 ± 2**			
Potassium (mEq/L) (2.9–5.3)	HF	4.3 ± 0.2	**4.5 ± 0.2**	**4.1 ± 0.2**	**4.0 ± 0.3**	*P* < 0.01	*P* = 0.24	*P* = 0.21
	CO	4.2 ± 0.3	**4.5 ± 0.2**	**4.0 ± 0.2**	**4.0 ± 0.1**			
	LF	4.3 ± 0.3	**4.5 ± 0.2**	**4.0 ± 0.3**	**4.1 ± 0.3**			
Phosphorus (mg/dL) (2.8–5.3)	HF^a^	4.7 ± 0.6	**4.2 ± 0.3**	**3.8 ± 0.5**	**3.7 ± 0.7^a^**	*P* < 0.01	*P* = 0.01	*P* = 0.99
	CO	4.4 ± 0.5	**3.9 ± 0.6**	**3.5 ± 0.7**	**3.3 ± 0.8^b^**			
	LF	4.3 ± 0.5	**3.9 ± 0.5**	**3.4 ± 0.7**	**3.2 ± 0.9^b^**			
Calcium (mg/dL) (9.3–11)	HF	10.4 ± 0.3	**10.2 ± 0.2**	10.2 ± 0.4	**10.1 ± 0.4**	*P* < 0.01	*P* = 0.17	*P* = 0.67
	CO	10.5 ± 0.3	**10.3 ± 0.3**	10.3 ± 0.3	**10.3 ± 0.3**			
	LF	10.4 ± 0.3	**10.2 ± 0.2**	10.2 ± 0.3	**10.2 ± 0.3**			
Magnesium (mg/dL) (1.4–2.0)	HF	1.6 ± 0.1	**1.5 ± 0.1**	**1.5 ± 0.1**	**1.5 ± 0.1**	*P* < 0.01	*P* = 0.07	*P* = 0.33
	CO	1.7 ± 0.1	**1.6 ± 0.1**	**1.5 ± 0.1**	**1.5 ± 0.1**			
	LF	1.6 **±** 0.1	**1.5 ± 0.1**	**1.5 ± 0.1**	**1.5 ± 0.1**			
Alk phos (U/L) (12–122)	HF^a^	28 ± 13^a^	**30 ± 12^a^**	28 ± 12^a^	28 ± 12^a^	*P* = 0.04	*P* < 0.01	*P* = 0.93
	CO	48 ± 26^b^	**49 ± 26^b^**	46 ± 25^b^	47 ± 25^b^			
	LF	54 ± 24^b^	**56 ± 24^b^**	53 ± 24^b^	52 ± 22^b^			
AST (U/L) (16–50)	HF	41 ± 13	43 ± 13	**45 ± 15**	44 ± 14	*P* < 0.01	*P* = 0.10	*P* = 0.98
	CO	35 ± 8	38 ± 9	**41 ± 14**	36 ± 7			
	LF	39 ± 9	42 ± 10	**44 ± 14**	41 ± 8			
ALT (U/L) (25–106)	HF	53 ± 17	55 ± 17	55 ± 17	53 ± 17	*P* = 0.07	*P* = 0.09	*P* = 0.13
	CO	51 ± 10	54 ± 11	53 ± 11	52 ± 10			
	LF	58 ± 16	61 ± 18	60 ± 17	59 ± 16			
Urea (mg/dL) (8–30)	HF	21 ± 3^b^	22 ± 3^b^	22 ± 3^b^	22 ± 3^b^	*P* = 0.13	*P* = 0.01	*P* = 0.99
	CO^a^	15 ± 3^a^	16 ± 3^a^	16 ± 3^a^	16 ± 3^a^			
	LF	20 ± 3^b^	20 ± 3^b^	21 ± 3^b^	20 ± 4^b^			
Creatinine (mg/dL) (0.5–1.3)	HF	1.1 ± 0.1	**1.2 ± 0.2**	**1.2 ± 0.2**	1.2 ± 0.2	*P* = 0.01	*P* = 0.18	*P* = 0.84
	CO	1.1 ± 0.1	**1.2 ± 0.1**	**1.2 ± 0.2**	1.2 **±** 0.2			
	LF	1.1 ± 0.2	**1.2 ± 0.2**	**1.2 ± 0.2**	1.2 ± 0.2			
Creat. kinase (U/L) (58–241)	HF	123 ± 46	**135 ± 42**	**134 ± 42**	132 ± 54	*P* = 0.05	*P* = 0.37	*P* = 0.68
	CO	106 ± 35	**116 ± 35**	**124 ± 62**	99 ± 35			
	LF	128 ± 64	**140 ± 90**	**144 ± 111**	141 ± 78			
Albumin (g/dL) (3.1–4.1)	HF	3.6 ± 0.2	**3.7 ± 0.2**	**3.7 ± 0.2**	3.6 ± 0.2	*P* < 0.01	*P* = 0.08	*P* = 0.72
	CO	3.7 ± 0.2	**3.8 ± 0.2**	**3.7 ± 0.2**	3.7 ± 0.2			
	LF	3.6 ± 0.1	**3.7 ± 0.2**	**3.7 ± 0.2**	3.6 ± 0.2			
Globulin (g/dL) (1.9–3.6)	HF	2.1 ± 0.3	**2.2 ± 0.3**	2.2 ± 0.4	2.2 ± 0.3	*P* < 0.01	*P* = 0.65	*P* = 0.87
	CO	2.1 ± 0.3	**2.1 ± 0.2**	2.1 ± 0.3	2.1 ± 0.2			
	LF	2.1 ± 0.2	**2.2 ± 0.2**	2.2 ± 0.3	2.1 ± 0.2			

*Bolded text indicates a difference from pre-exercise over time; ^a^In dietary groups represents a significant difference between groups (*P* < 0.05). ^a,b^Across time points represent significant differences (*P* < 0.05) between groups at individual times with similar or no superscripts signifying no differences*.

There were significant diet effects on small number of chemistry parameters. The CO diet resulted in overall lower serum urea nitrogen concentrations (*P* = 0.01), while the HF diet resulted in lower alkaline phosphatase concentrations (*P* < 0.01). The HF dietary treatment also resulted in significantly higher phosphorus (*P* = 0.01) and chloride concentrations (*P* < 0.01) than the CO or LF dietary treatments. At 20-min post-exercise, serum phosphorus was significantly higher in dogs that had consumed the HF diet (*P* = 0.04).

There were modest effects of exercise observed with serum phosphorus concentrations being significantly lower in all diet groups 10-min post-exercise and at 20-min post-exercise (*P* < 0.01). Concurrently, serum magnesium showed a similar decrease with significantly lower concentrations at immediately post, 10 and 20 min of recovery compared to pre-exercise concentrations (*P* < 0.05). Creatine kinase and creatinine also showed similar more transient increased in serum concentrations post-exercise, which returned to pre-exercising concentrations by 20 min of recovery (*P* < 0.01). Albumin and globulin also showed transient increases immediately post and 10 min after exercise when compared to pre-exercise values (*P* < 0.01).

### Lactate, Glucose, Non-Esterified Fatty Acids, and Serum Cortisol

There were no diet and exercise interactions or significances observed for glucose (*P* = 0.05), lactate (*P* = 0.26), non-esterified fatty acids (*P* = 0.76), or cortisol (*P* = 0.26) (Table 4).

**Table 4:** **Mean and SD for glucose, non-esterified fatty acid, and cortisol; pre-exercise, post-exercise, 10-min post exercise and 20-min post exercise for three dietary groups (*n* – 17) during feeding a high-fat high-protein diet (HF), high corn oil fat low protein diet (CO), and a low-fat high-protein diet (LF)**.

Metab/hormones	Diet	Pre	Post	Post 10 min	Post 20 min	Time	Diet	Diet × time
Glucose (mg/dL) (60–120)	HF	98 ± 9	100 ± 10	99 ± 9	100 ± 10^b^	*P* < 0.01	*P* = 0.05	*P* = 0.16
	CO^a^	96 ± 10	103 ± 11	**105 ± 10**	**108 ± 7^a^**			
	LF	96 ± 10	103 ± 10	**103 ± 11**	102 ± 9^b^			
Lactate (mmol/L) (0.5–2)	HF	0.50 ± 0.26	**1.35 ± 0.82**	**1.36 ± 0.83**	**1.17 ± 0.62**	*P* < 0.01	*P* < 0.01	*P* = 0.26
	CO	0.51 ± 0.21	**1.42 ± 1.25**	**1.37 ± 1.16**	**1.10 ± 0.84**			
	LF^a^	0.56 ± 0.16	**1.98 ± 1.08^a^**	**1.93 ± 1.06**	**1.63 ± 0.87**			
NEFA (mg/dL) (0–1)	HF	0.48 ± 0.16	**1.15 ± 0.39**	**1.14 ± 0.47**	**0.73 ± 0.35**	*P* < 0.01	*P* = 0.72	*P* = 0.76
	CO	0.52 ± 0.23	**1.21 ± 0.41**	**1.10 ± 0.37**	**0.83 ± 0.35**			
	LF	0.38 ± 0.24	**1.21 ± 0.42**	**1.11 ± 0.44**	**0.74 ± 0.39**			
Cortisol (μg/mL) (1–4)	HF^a^	1.22 ± 0.52	**2.93 ± 2.31^a^**	**2.54 ± 1.89^a^**	**1.94 ± 1.52^a^**	*P* < 0.01	*P* < 0.01	*P* = 0.26
	CO	1.63 ± 1.15	**3.52 ± 2.21^a,b^**	**2.91 ± 1.79^a,b^**	**2.25 ± 1.40^a,b^**			
	LF	1.25 ± 0.37	**4.58 ± 2.42^b^**	**4.30 ± 2.67^b^**	**3.56 ± 2.36^b^**			

*Bolded text indicates a difference from pre-exercise over time; ^a^In dietary groups represents a significant difference between groups (*P* < 0.05). ^a,b^Across time points represent significant differences (*P* < 0.05) between groups at individual times with similar or no superscripts signifying no differences*.

There were significant effects of diet observed for glucose (*P* = 0.05) with mean concentrations being higher for the CO diet compared to LF and HF. Dogs consuming the CO diet showed a mild, yet significant increase in serum glucose at 20-min post exercise (*P* = 0.01). Mean serum lactate (*P* < 0.01) was significantly higher for dogs in the LF treatments compared to the HF and CO treatments. Lactate in dogs consuming the LF diet was higher than either the CO or HF diets post-exercise (*P* < 0.05). Serum cortisol concentrations were lower for dogs on the HF diet (*P* < 0.01). Dogs consuming the HF diet had significantly lower serum cortisol concentrations at all post-exercise time points than the LF-fed dogs (*P* < 0.01).

The effects of exercise over time showed significances across all four parameters tested. All test subjects had increased serum lactate concentration (*P* < 0.01) immediately post-exercise and thereafter when compared to pre-exercise (*P* < 0.01). Serum non-esterified fatty acids were increased in all dogs regardless of dietary group immediately post-exercise and remained elevated compared to the pre-exercise values (*P* < 0.01). Serum cortisol concentrations were higher immediately post-exercise in all groups (*P* < 0.01). They remained significantly increased at 10- and 20-min post-exercise, though gradually returning toward baseline values.

### Core and Rectal Body Temperatures

**Figure 1:**
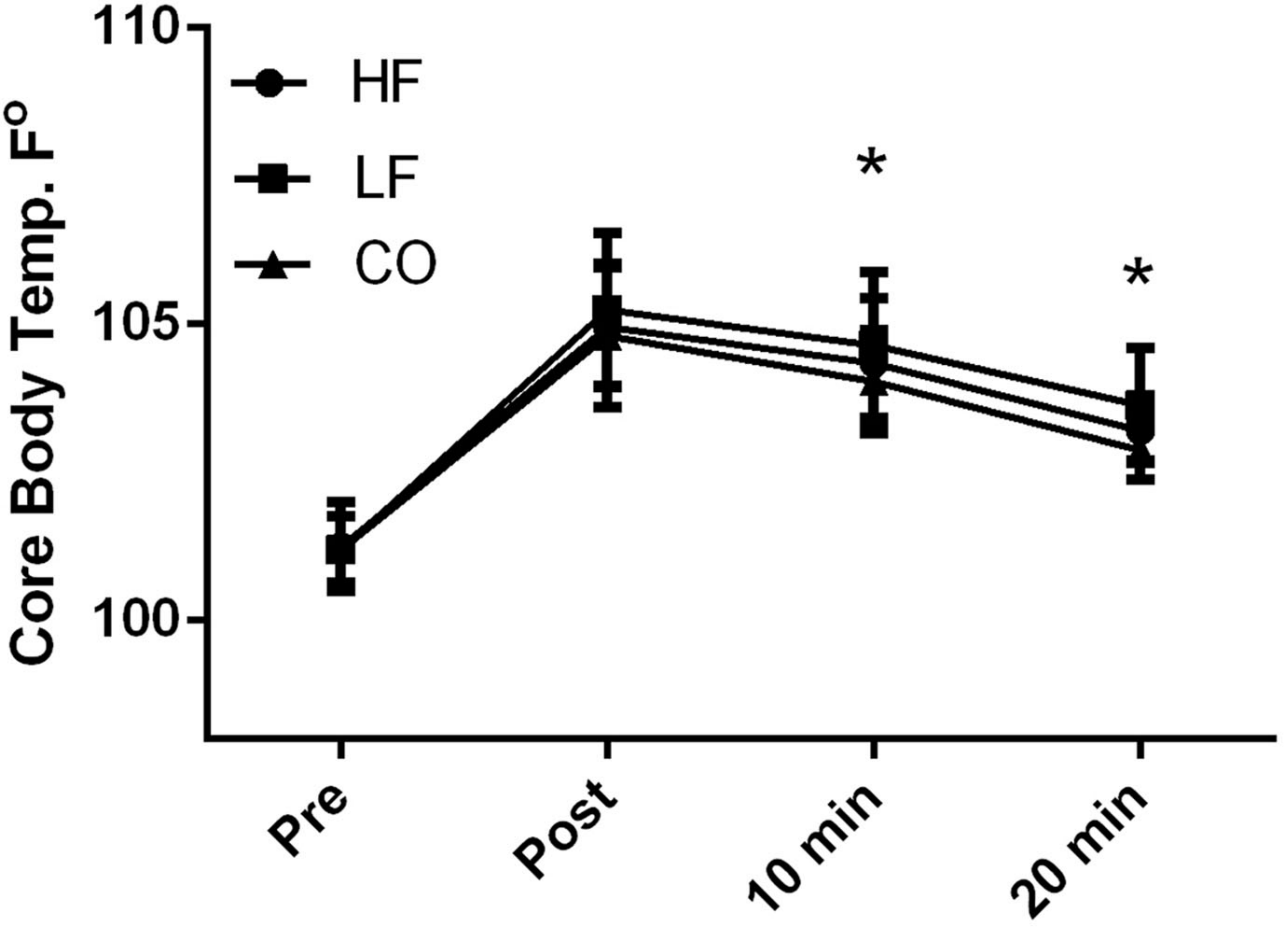
**Mean and SD of core thermister readings before immediately after, 10 min of recovery and 20 min of recovery in high-fat (HF), low-fat (LF), and corn oil (CO) treatments**. Significances between dietary groups represented astericks showing CO treatment having lower temperatures than the LF group (10 min *P* = 0.04; 20 min *P* = 0.02). Time was significant between each time point for all groups (*P* < 0.01).

There were no diet and exercise interactions observed for core body temperature (*P* = 0.32); however, there was an effect of diet observed with overall lower core temperatures (*P* < 0.01) in the dogs on the CO diet. Similarly, dogs consuming the CO diet had significantly (*P* < 0.05) lower core temperature 10 and 20 min after exercise when compared to the LF ration (Figure 1). Pre-exercise the core body temperatures were not significantly different (HF – 101.2 ± 0.8°F; LF – 101.2 ± 0.6°F; CO – 101.1 ± 0.6°F) with significant rises post-exercise (*P* < 0.01) across all groups (HF – 105.0 ± 1.0°F; LF – 105.2 ± 1.3°F; 104.8 ± 1.2°F). At 10-min post-exercise and 20-min post-exercise, core body temperatures were still elevated compared to pre-exercise (10-min post: HF – 104.3 ± 1.1°F; LF – 104.7 ± 1.2°F; CO – 104.0 ± 0.8°F and 20-min post: HF 103.2 ± 0.5°F; LF – 103.6 ± 1.0°F; CO –102.9 ± 0.5°F).

**Figure 2:**
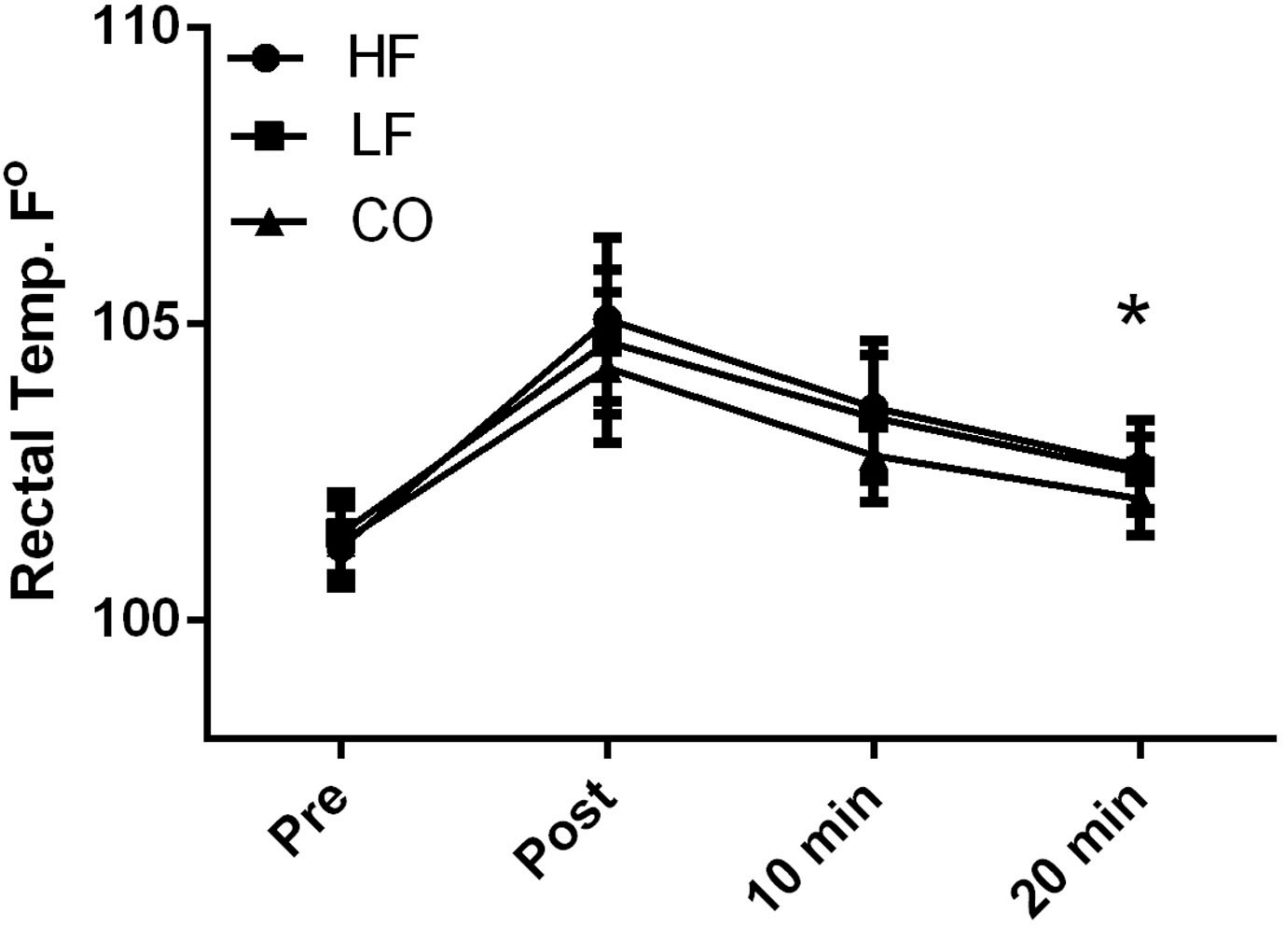
**Mean and SD of rectal thermometer readings before immediately after, 10 min of recovery and 20 min of recovery in high-fat (HF), low-fat (LF), and corn oil (CO) treatments**. Significances between dietary groups represented asterisks showing CO treatment having lower temperatures than the HF group (*P* = 0.03). Time was significant between each time point for all groups (*P* < 0.01).

There were no diet and exercise interactions observed for rectal body temperature (*P* = 0.45); however, there was an effect of diet observed with overall lower rectal temperatures (*P* < 0.01) in the dogs on the CO diet. Dogs consuming the CO diet had significantly (*P* < 0.05) lower rectal temperature 20-min post-exercise than dogs consuming the HF ration (Figure 2). Rectal temperature was significantly (*P* < 0.01) higher immediately post-exercise in all groups, relative to baseline (Pre: HF – 101 ± 0.7°F; LF – 101.5 ± 0.7°F; CO – 101.3 ± 0.7°F and Post: HF 105.1 ± 1.4°F; LF – 104.7 ± 1.3°F; CO – 104.3 ± 1.3°F). This parameter remained significantly elevated 10-min post-exercise (HF – 102.6 ± 0.8°F; LF – 103.4 ± 1.1°F; CO – 102.8 ± 0.8°F). By 20-min post-exercise, temperatures approached baseline values and were no longer statistically significant from baseline (HF – 102.6 + 0.8°F; LF – 102.5 + 0.6°F; CO – 102.1 + 0.6°F).

## Discussion

The effects of diet and exercise and complete blood count, serum biochemistry, blood gases, and vital parameters appear to be subtle in our study, but relevant, considering dietary change and exercise in conditioned Labradors trained for detection activities has never been assessed in a controlled manner. Our leading hypothesis was that low-protein diets would result in alterations in serum protein biochemistry, since dietary protein may play a role in maintaining appropriate red blood cell indices and it has been noted that performance dogs require 24% of their ME as high quality dietary protein to maintain performance capabilities (1–3, 7). In all of the diets used in our study, the protein base was from poultry and varied between 18 and 27% ME proving to be adequate for the dogs enrolled in this study under this type of daily exercise pattern. Our results suggest that the red blood cell counts and volumes were slightly lower universally during CO treatment with universal mild hemoconcentration occurring during exercise that was significant with modest lymphocyte decreases due to exercise (15, 16). This slightly lower red cell count with CO treatment warrants trepidation regarding feeding a lower protein diet (18% ME) due to potential long-term physiological consequences, although in this 12-week trial on each of the diets the mild alterations did not seem to affect performance according to handlers.

Serum albumin is possibly a more sensitive marker of protein deficiency than red blood cell counts. Reynolds and colleagues suggested that albumin was highest in dogs fed 32% ME as protein, while lowest in dogs fed 18% ME protein during a 16-week dietary trial (7). Serum albumin is considered a long-term marker of protein sufficiency in normal healthy dogs and we expected that serum albumin would decrease in the dogs being fed the 18% ME protein diet; however, it did not decrease over the 12-week time period. This suggests that the protein in the diet was adequate at 18% of the ME for this group of conditioned dogs; yet this feeding trial was relatively short. Longer periods of feeding may be necessary to see decreases in albumin.

Furthermore, unlike Reynolds who observed more musculoskeletal injuries when dogs were fed 18% ME (7), no musculoskeletal injuries or performance deficits during detection or conditioning activities were noted in the health records among handlers who were blinded to diet. The dogs in our study could have been consuming more protein per kilogram body weight when on the 18% ME diet than dogs in the Reynolds study; however, when examining the median and range of caloric intake from both studies, they were similar based on kilograms of body weight. Hence, we postulate that 18% ME may be adequate for low-intensity long-duration activity, but longer term feeding trials are warranted.

Further examination of the blood chemistry parameters showed a distinct change in urea concentrations due to protein consumption. Dogs fed 18 versus 27% had globally decreased serum urea nitrogen concentrations suggesting less protein metabolism in the 18% ME group. One limitation of our study was the lack of lean body mass measurement, which may have changed during the feeding of 18% ME protein, but based on handler observations this lower protein consumption did not alter performance and may actually enhance olfactory performance when tested during the same time period (13).

Much like the changes observed metabolically due to the protein differences in the diet there were some changes observed due to the fat and carbohydrate differences in ME. For the LF group, we observed a rise in serum lactate during exercise when compared to the two groups being fed 57% ME as fat. This is consistent with observations in other exercise models where dogs being fed a high-fat diet tended to have lower serum lactate (17). Consistent with the metabolic demands of exertion in the well-conditioned endurance athlete (4, 18), serum non-esterified fatty acid concentration was significantly different immediately post-exercise relative to pre-exercise values, and gradually recovered toward pre-exercise values during the ensuing 20 min of recovery for all groups.

Probably, the most interesting results from our study revolve around the vital signs of core temperature and rectal temperature. Subjects consuming the corn oil top-dressed diet showed globally lower core and rectal temperatures post-exercise and quicker thermal recovery with a significant difference at specific time points during recovery. The true impact of this in clinical/field situations remains to be fully elucidated; however, reduction of thermal stress by diet formulation represents a simple and inexpensive opportunity to potentially improve the performance of detection dogs. Formulation of rations for detection dogs for further study suggest that protein and fat (as well of fat sourcing) may be worthwhile and begs for further field exploration.

The thermal changes do not seem to be reflected in the venous blood gas changes; however, arterial blood gases would have been more appropriate for interpretation of gas exchange. It is clear that the venous pCO_2_ was low in all dogs prior to exercise, which was likely from panting due to the excitement associated with the activity, further confounding any changes associated with exercise and diet (19). Corn oil consumption resulted in modestly higher venous pCO_2_ than the low fat group at 10-min post-exercise. It may be that dogs consuming corn-oil experience less thermal stress during exertion, recover from it more efficiently, and may be less driven to alter respiratory pattern (panting) to recover normal body temperature retaining slightly more CO_2_; but this is highly speculative base on our venous blood gas results.

The significant reduction of serum phosphorus concentration in the low-fat diet group appears perplexing; however, again this group was less conditioned for fat metabolism, hence more carbohydrate metabolism as an energy source was utilized. Glucose utilization as a major fuel to meet metabolic demands during this bout of exercise requires phosphorus during glycogenolysis to form glucose-1-phosphate and glucose-6-phosphate potentially leading to an intracellular phosphorus flux during exercise as observed in other exercising canine studies (20, 21). The reason for no dietary differences in phosphorus influx is likely due to the exercise bout being well within the range the time range for carbohydrate oxidation, which relies on either glycogenolysis and to a lesser degree protein or fat metabolism (22). In addition, the CO group had similarly lower serum phosphorus to the LF group, which might be a reflection of overall lower phosphorus in the diet due to corn oil dilution of overall phosphorus intake in this group.

Cortisol has been used as an indicator of stress and is often elevated in acute exercise stress models (15, 23). The dogs consuming 57% of ME as fat in a complete diet had a statistically and physiologically significant reduction in serum cortisol concentration. We cannot explain why this did not happen when the dogs consumed kibble top-dressed with corn oil, with the possible exception that the latter diet was obviously not properly balanced following the addition of the corn oil, with the HF diet often having higher concentrations of many vitamin and mineral; or possibly that the high polyunsaturated fat in the corn oil diet can influence serum cortisol (13). Whether these modest differences in cortisol influence cellular proteolysis or glycogenesis/glycogenolysis remain to be elucidated, yet there were no changes in rudimentary serum glucose or non-esterified fatty acids that could be explained by the modest alterations in serum cortisol other than a mild elevation in serum glucose in the CO group at 20 min of recovery that was not seen in the LF or the HF group. Further study of nutritional influences on cortisol secretion and synthesis are needed to better understand these findings. Interestingly, serum alkaline phosphatase was significantly lower in dogs consuming the HF diet. We posit that the lower alkaline phosphatase of dogs consuming a nutritionally complete and balanced high-fat diet is consistent with their lower serum cortisol concentration, representing a basis for reduced metabolic stress.

## Conclusion

For working dogs, the lifetime cost of feed, even if specially formulated, represents a trivial fraction of the monetary investment in training and purchase. Formulation of a nutritionally complete and balance ration with 18% of ME as protein and polyunsaturated fat as the primary fat source may aid in post-exercise thermoregulation, support maintenance of body weight, albumin, and hemogram profiles; and offer potential improvements in challenging work situations during short-term feeding (12 weeks). These data suggest that diet can affect baseline cortisol secretion and ameliorate dissipation of body heat and further study and optimization of this dietary strategy may be worthwhile for detection dogs.

## Author Contributions

JW, TA, and RG postulated the experimental design. PH, JW, RG, TA, and JO performed work associated with this study. RG, JW, JO, and DF performed statistical analysis and prepared manuscript. All authors reviewed manuscript upon submission.

## Conflict of Interest Statement

The authors declare that the research was conducted in the absence of any commercial or financial relationships that could be construed as a potential conflict of interest.

## Footnotes

^1^Low Fat (LF) diet: Royal Canin 2500, Armitage, France

^2^High Fat (HF) diet: Royal Canin 4800. Armitage, France

^3^Corn oil, ACH Food Companies, Cordova, TN, USA

^4^Jog a dog Treadmill, Jog a Dog LLC, Ottawa Lake, MI, USA

^5^Core Temp Sensor, HQ Inc., Palmetto, FL, USA

^6^Digital Rectal Thermometer, Becton Dickenson, Franklin Lakes, NJ, USA

^7^iSTAT, Abbott Care Inc., Princeton, NJ, USA

^8^CG8 cartridge, Abbott Care Inc., Princeton, NJ, USA

^9^Advia 120, Siemens Corp., Washington, DC, USA

^10^Hitachi 911, Roche Diagnostics, Risch-Rotkreuz, Switzerland

^11^JMP 12.0, SAS Institute, Cary, NC, USA

^12^Graph pad Prism, LaJolla, CA, USA

## Abbreviations

BCS, body condition score; ME, metabolic energy.
